# *CYP4V2* rs56413992 C > T was associated with the risk of coronary heart disease in the Chinese Han population: a case–control study

**DOI:** 10.1186/s12920-023-01737-y

**Published:** 2023-12-08

**Authors:** Kang Huang, Tianyi Ma, Qiang Li, Zanrui Zhong, Yilei Zhou, Wei Zhang, Ting Qin, Shilin Tang, Jianghua Zhong, Shijuan Lu

**Affiliations:** 1 Department of Cardiology, Central South University Xiangya School of Medicine Affiliated Haikou Hospital, No. 43, Renmin Avenue, Haikou, Hainan China; 2 School of Medicine, Jingchu University of Technology, Jingmen, Hubei China

**Keywords:** Coronary heart disease, *CYP4V2*, Polymorphisms, Chinese Han population

## Abstract

**Purpose:**

The research aimed to detect the association between single nucleotide polymorphisms (SNPs) in *CYP4V2* gene and coronary heart disease (CHD) risk.

**Methods:**

This case–control study included 487 CHD subjects and 487 healthy individuals. Logistic regression was performed to analyze the connection between five SNPs in *CYP4V2* (rs1398007, rs13146272, rs3736455, rs1053094, and rs56413992) and CHD risk, and odds ratios (ORs) with 95% confidence intervals (CIs) were calculated to evaluate the connection.

**Results:**

As a result, we found that rs56413992 T allele (OR = 1.36, 95% CI = 1.09–1.70, *p* = 0.007) and CT genotype (OR = 1.40, 95% CI = 1.06–1.83, *p* = 0.017) were significantly associated with an increased risk of CHD in the overall analysis. Precisely, rs56413992 was linked to an elevated risk of CHD in people aged > 60, males, smokers and drinkers. The study also indicated that rs1398007 was linked to an increased CHD risk in drinkers. In addition, rs1053094 was correlated with a decreased risk of CHD complicated with diabetes mellitus (DM), and rs1398007 was correlated with a decreased risk of CHD complicated with hypertension (HTN).

**Conclusion:**

This study was the first to experimentally demonstrate that *CYP4V2* rs56413992 was associated with the risk of CHD, which will provide a certain reference for revealing the pathogenesis of CHD.

**Supplementary Information:**

The online version contains supplementary material available at 10.1186/s12920-023-01737-y.

## Introduction

Coronary heart disease (CHD) is one of the main causes of morbidity and death in cardiovascular diseases worldwide, and it is also one of the major diseases that threaten human life and health [1]. According to statistics in 2019, about 8.88 million people died of CHD in the world, and the incidence is still rising. Studies have shown that CHD is a common disease caused by coronary atherosclerosis, and its main feature is lipid deposition in the arterial intima [2, 3]. Specifically, when the body’s lipid metabolism is abnormal, abnormal accumulation of lipid will occur in the arterial intima, which promotes atherosclerotic lesions, causing reduced or blocked blood flow in coronary blood flow, and ultimately leading to CHD [4]. It has been reported that the etiology of CHD is multifactorial, including environmental and genetic factors, as well as their combined effect [5–7]. In recent years, a larger number of studies have suggested that genetic factors, especially single nucleotide polymorphisms (SNPs), play a significant role in the mechanism of CHD [8–10]. Thus, it is essential to study CHD-related gene polymorphisms.

Cytochrome P450 (P450), an important enzyme of oxidative metabolism, plays a recognized part in the metabolism of endogenous compounds and the metabolic clearance of drugs and other exogenous substances [11]. *CYP4V2* is a member of the cytochrome P450 family 4 [11]. *CYP4V2* gene, located in the chromosomal region 4q35, contains 11 exons and encodes the protein composed of 525 amino acids [12]. Studies have shown that *CYP4V2* is a selective ω-hydroxylase for saturated medium-chain fatty acids [13]. Currently, although *CYP4V2* has been proven to be involved in lipid metabolism, its role in the pathogenesis of CHD remains unclear [14].

**Fig. 1:**
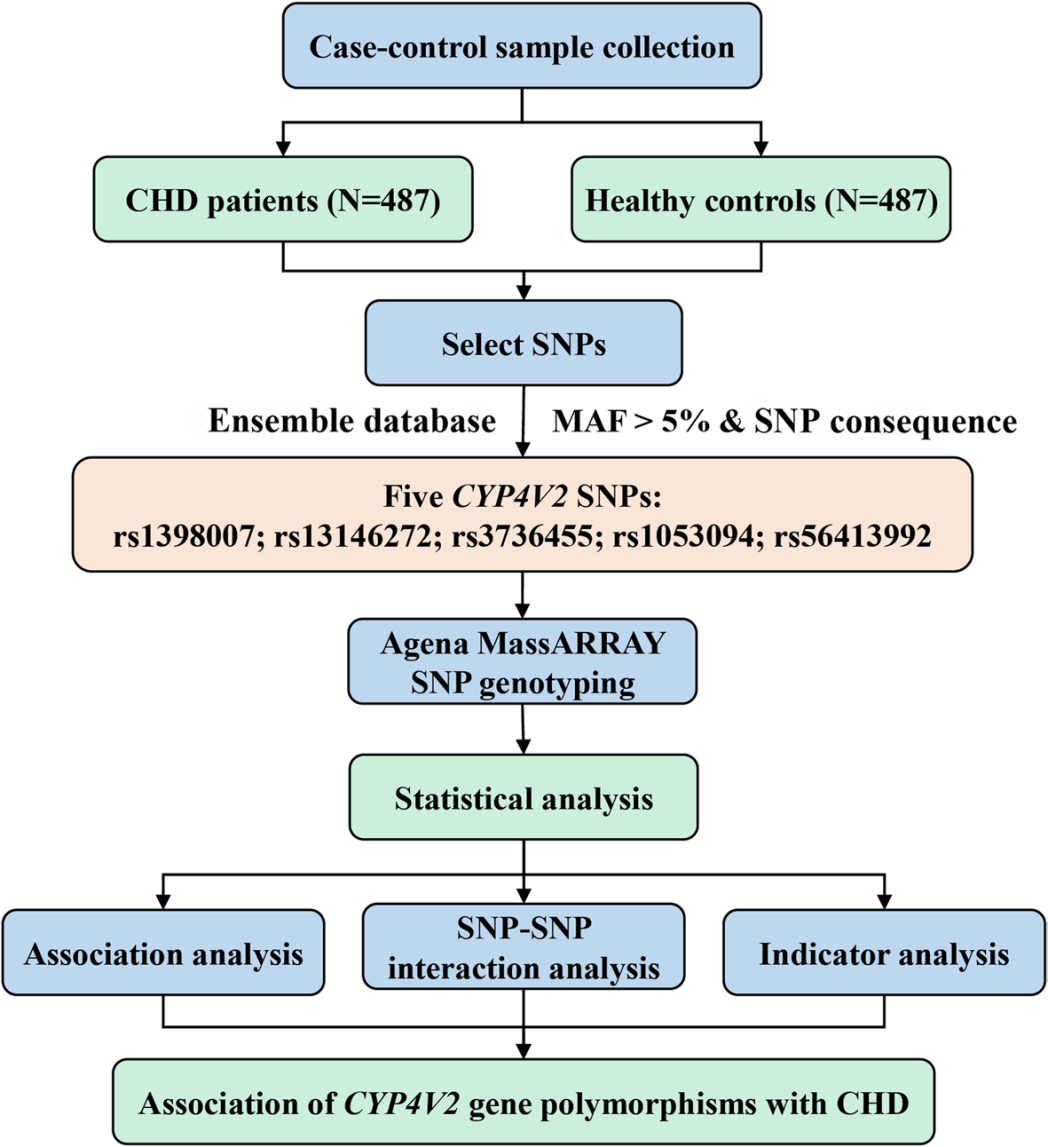
Flowchart of a study on the relationship between *CYP4V2* polymorphisms and CHD risk

Therefore, this research aimed to investigate the connection of *CYP4V2* rs1398007, rs13146272, rs3736455, rs1053094 and rs56413992 with the risk of CHD in the Chinese Han population, to provide reference value for the diagnosis, prevention and prognosis of CHD. The research flow chart of this study was shown in Fig. 1.

## Material and methods

### Participants

On the basis of G*power 3.1.9.7 software, we estimated the sample size of the case group and the control group through an independent sample t-test. The parameters were set as: Tail = 2, Effect size = 0.20, α = 0.05, Power = 0.85, Allocation ratio N2/N1 = 1. Ultimately, this case–control research recruited 487 CHD subjects from the department of cardiology of Central South University Xiangya School of Medicine Affiliated Haikou Hospital and 487 healthy individuals from the medical examination center of the hospital. They are all ethnic Han Chinese. The diagnosis of CHD patients was based on the comprehensive examination of clinical symptoms, medical history, and diagnostic tests, including electrocardiograms and coronary angiography. The inclusion criterion for the CHD group was: single-vessel stenosis > 70% or multi-vessel stenosis > 50% confirmed by coronary angiography. Patients with congenital heart disease, acute myocardial infarction, cardiomyopathy, malignant tumor and chronic inflammatory disease were excluded. The inclusion criterion for the healthy control group was: (1) healthy individuals without cardiovascular diseases confirmed by physical examination; (2) unrelated subjects matched for age and gender with patients with CHD; (3) no family history of cardiovascular and cerebrovascular diseases; (4) no diabetes and hypertension. Besides, basic information about the participants, including age, gender, smoking and alcohol consumption status, complicated with DM and HTN and clinical parameters-total cholesterol (TC), triglyceride (TG), high-density lipoprotein cholesterol (HDL-C) and low-density lipoprotein cholesterol (LDL-C) was collected from standard questionnaires and medical records. HTN is defined as systolic blood pressure ≥ 140 mmHg and diastolic blood pressure ≥ 90 mmHg, or the patient is receiving antihypertensive medication. DM is defined as fasting venous plasma glucose level ≥ 7.0 mmol/L, random venous plasma glucose level ≥ 11.1 mmol/L, or the patient is being treated with hypoglycemic drugs or insulin. Also, we divided the participants into smokers, non-smokers, drinkers and non-drinkers according to their smoking and drinking habits, respectively. All participants were unrelated Chinese Han population. This research was ratified by the Central South University Xiangya School of Medicine Affiliated Haikou Hospital ethics committee (ZY-IRB-FOM-063), and written informed consent was obtained from each subject.

### SNP selection and genotyping

In this study, the following processes were used to select SNPs in *CYP4V2* gene: (1) 18,005 loci of *CYP4V2* were found in the Ensemble database (https://asia.ensembl.org/index.html); (2) There were 361 variants with minor allele frequency (MAF) greater than 5% in the general population published in the 1000 Genome Project database; (3) Twenty-five variants were obtained according to their functional categories (5'-UTR, 3'-UTR, missense and synonymous); (4) After consulting relevant literature [15–17], five variants (rs1398007, rs13146272, rs3736455, rs1053094 and rs56413992) were selected to continue to evaluate the correlation with the risk of CHD.

For each subject, 5 mL of peripheral venous blood sample was collected and stored in EDTA anticoagulant tubes at 4°C for DNA extraction by GoldMag genomic DNA purification kit (GoldMag Co Ltd., Xi’an, China). Primers for PCR amplification were designed using Agena Bioscience Assay Design software, and the information of PCR primers was shown in Supplementary Table 1. SNPs were then genotyped using the Agena MassARRAY iPLEX platform (Agena Bioscience Inc., CA, USA). Finally, Agena Bioscience TYPER 4.0 software was applied to manage and analyze the results of SNP genotyping.

### Statistical analyses

SPSS 22.0 was used for statistical analysis in this study. All tests were two-sided, and *p* < 0.05 was considered statistically significant. Independent sample t-test and Chi-square test were carried out to analyze the sample characteristics of continuous variables and categorical variables, respectively. The chi-square test was used to check whether the genotype distribution of the five loci in the control group was consistent with the HWE. Logistic regression was used to analyze the association between SNPs and CHD risk, and ORs with their corresponding 95% CIs were used to evaluate these association. Stratified analysis was also performed according to age, gender, smoking and drinking status, CHD complicated with DM, and CHD complicated with HTN. In addition, false positive report probability (FPRP) analysis was also performed to verify the reliability of the significant results in this study. Haploview software 4.2 was used to calculate the degree of linkage among these SNPs based on the linkage disequilibrium (LD) map. Multifactor dimensionality reduction (MDR) method was used to analyze SNP-SNP interactions and predict their effect on CHD risk.

## Results

### Basic clinical characteristics of study participants

The study population consisted of 487 CHD subjects and 487 healthy individuals. The basic information on CHD cases and healthy controls was shown in Table 1. The average age of CHD subjects and healthy individuals were 61.91 ± 10.18 years and 60.82 ± 9.03 years, respectively, which illustrated the age distribution of the two groups matched (*p* = 0.078). In addition, there was no significant difference in the distribution of gender, smoking and drinking status between the CHD group and the control group (*p* = 0.733, *p* = 0.847, *p* = 0.189). However, there were statistically significant differences in the distribution of TC, TG, HDL-C and LDL-C between the CHD group and the control group.

**Table 1:** Basic characteristics of the study population

Parameter	Case (*n* = 487)	Control (*n* = 487)	*p*
**Age (years, Mean ± SD)**	61.91 ± 10.18	60.82 ± 9.03	0.078^a^
> 60	265(54.4%)	262(53.8%)	
≤ 60	222(45.6%)	225(46.2%)	
**Gender, n (%)**			0.733^b^
Male	324(66.5%)	329(67.6%)	
Female	163(33.5%)	158(32.4%)	
**Smoke**			0.847^b^
Yes	256(52.6%)	259(53.2%)	
No	231(47.4%)	228(46.8%)	
**Drink**			0.189^b^
Yes	180(37%)	200(41.4%)	
No	307(63%)	287(58.9%)	
**Serum lipid levels**			
TC	4.07 ± 1.04	4.76 ± 0.91	***p* < 0.001**^**a**^
TG	1.62 ± 1.01	1.85 ± 1.48	**0.007**^**a**^
HDL-C	1.11 ± 0.26	1.17 ± 0.37	**0.020**^**a**^
LDL-C	2.43 ± 0.94	2.61 ± 0.73	**0.002**^**a**^
**Complicated with DM**			
Yes	143(29.4%)		
No	344(70.6%)		
**Complicated with HTN**			
Yes	303(62.3%)		
No	184(37.8%)		

*SD* standard deviation, *n* number, *TC* total cholesterol, *TG* triglyceride, *HDL-C* high-density lipoprotein cholesterol, *LDL-C* low-density lipoprotein cholesterol, *DM* diabetes mellitus, *HTN* Hypertension

^a^*p* values were calculated by Student’s *t*-tests

^b^*p* values were calculated from two-sided chi-square test

### Basic information and allele frequency distribution of CYP4V2 SNPs

The basic information of SNPs in *CYP4V2* were shown in Table 2. The genotype distribution of rs1398007, rs13146272, rs3736455, rs1053094 and rs56413992 in the control group were in accordance with HWE. Among them, rs56413992-T allele in *CYP4V2* was associated with a 1.36-fold increased risk of CHD (OR = 1.36, 95% CI = 1.09–1.70, *p* = 0.007).

**Table 2:** Basic information and allele frequency distribution of *CYP4V2* SNPs

SNP_ID	Gene	Chr: Position	Consequence	Allele	MAF		HWE *p*-value	OR (95% CI)	*p*
					**Case**	**Control**			
rs1398007	*CYP4V2*	4: 186,191,678	5'-UTR	C > T	0.324	0.294	0.585	1.16(0.95–1.40)	0.141
rs13146272	*CYP4V2*	4: 186,199,057	Missense	C > A	0.390	0.383	0.632	1.03(0.86–1.24)	0.753
rs3736455	*CYP4V2*	4: 186,201,165	Synonymous	T > G	0.394	0.394	0.448	1.00(0.84–1.20)	0.984
rs1053094	*CYP4V2*	4: 186,211,877	3'-UTR	T > A	0.367	0.338	0.480	1.13(0.94–1.36)	0.195
rs56413992	*CYP4V2*	4: 186,213,040	3'-UTR	C > T	0.229	0.179	1.000	1.36(1.09–1.70)	**0.007**

*SNP* single nucleotide polymorphism, *Chr* Chromosome, *MAF* minor allele frequency, *HWE* Hardy–Weinberg equilibrium, *OR* odds ratio, *95% CI* 95% confidence interval

Bold values indicated that the *p*-value was statistically significant

*p*-value was calculated with Person’s chi-square test

### The overall analysis of the relationship between CYP4V2 SNPs and CHD risk

**Fig. 2:**
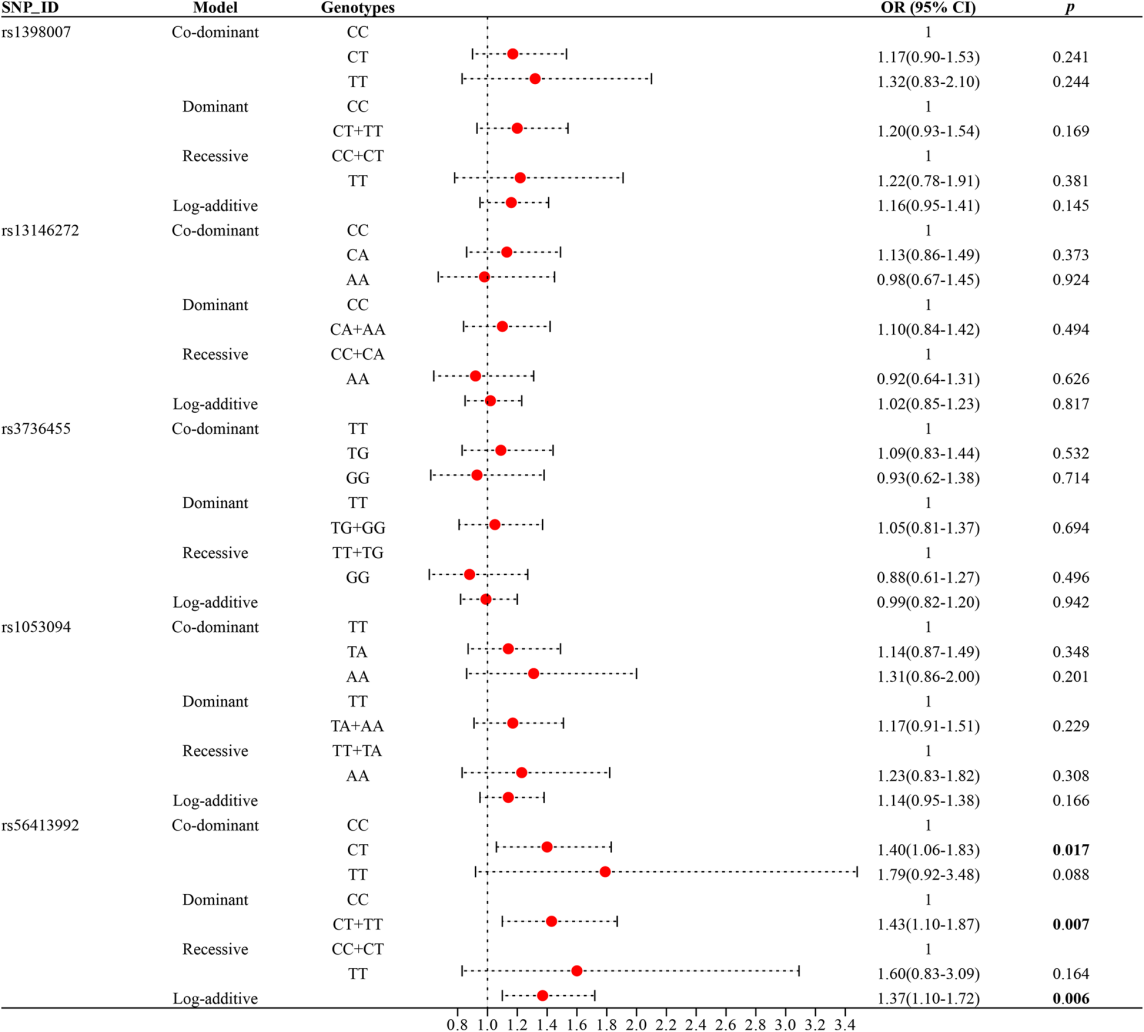
Forest map of the overall analysis of the relationship between *CYP4V2* polymorphisms and CHD risk

Adjusting for potential confounders, we performed logistic regression analysis based on co-dominant, dominant, recessive, and additive models, and the results showed that rs56413992 was significantly associated with an increased risk of CHD (Table 3). Specifically, in the heterozygous model, the CT genotype was associated with a 1.40-fold increased risk of CHD compared with the CC genotype (OR = 1.40, 95% CI = 1.06–1.83, *p* = 0.017). In addition, rs56413992 was also associated with an increased risk of CHD in dominant and log-additive models (dominant model: OR = 1.43, 95% CI = 1.10–1.87, *p* = 0.007; log-additive model: OR = 1.37, 95% CI = 1.10–1.72, *p* = 0.006). However, no association of rs1398007, rs13146272, rs3736455 and rs1053094 with CHD risk was observed. The forest diagram of the overall analysis of the relationship between *CYP4V2*polymorphisms and CHD risk was shown in Fig. 2.

**Table 3:** Association of *CYP4V2* gene polymorphisms with CHD risk in an overall analysis

SNP_ID	Model	Genotypes	Case	Controls	Adjusted	
					**OR (95% CI)**	***p***
**rs1398007**	Co-dominant	CC	218	240	1	
		CT	22	208	1.17(0.90–1.53)	0.241
		TT	47	39	1.32(0.83–2.10)	0.244
	Dominant	CC	218	240	1	
		CT + TT	269	247	1.20(0.93–1.54)	0.169
	Recessive	CC + CT	440	448	1	
		TT	47	39	1.22(0.78–1.91)	0.381
	Log-additive	—	—	—	1.16(0.95–1.41)	0.145
**rs13146272**	Co-dominant	CC	177	188	1	
		CA	239	225	1.13(0.86–1.49)	0.373
		AA	70	74	0.98(0.67–1.45)	0.924
	Dominant	CC	177	188	1	
		CA + AA	309	299	1.10(0.84–1.42)	0.494
	Recessive	CC + CA	416	413	1	
		AA	70	74	0.92(0.64–1.31)	0.626
	Log-additive	—	—	—	1.02(0.85–1.23)	0.817
**rs3736455**	Co-dominant	TT	168	174	1	
		TG	254	240	1.09(0.83–1.44)	0.532
		GG	65	71	0.93(0.62–1.38)	0.714
	Dominant	TT	168	174	1	
		TG + GG	319	311	1.05(0.81–1.37)	0.694
	Recessive	TT + TG	422	414	1	
		GG	65	71	0.88(0.61–1.27)	0.496
	Log-additive	—	—	—	0.99(0.82–1.20)	0.942
**rs1053094**	Co-dominant	TT	192	209	1	
		TA	233	225	1.14(0.87–1.49)	0.348
		AA	62	52	1.31(0.86–2.00)	0.201
	Dominant	TT	192	209	1	
		TA + AA	259	277	1.17(0.91–1.51)	0.229
	Recessive	TT + TA	425	434	1	
		AA	62	52	1.23(0.83–1.82)	0.308
	Log-additive	—	—	—	1.14(0.95–1.38)	0.166
**rs56413992**	Co-dominant	CC	288	326	1	
		CT	175	144	1.40(1.06–1.83)	**0.017**
		TT	24	15	1.79(0.92–3.48)	0.088
	Dominant	CC	288	326	1	
		CT + TT	199	159	1.43(1.10–1.87)	**0.007**
	Recessive	CC + CT	463	470	1	
		TT	24	15	1.60(0.83–3.09)	0.164
	Log-additive	—	—	—	1.37(1.10–1.72)	**0.006**

*SNP* single nucleotide polymorphism, *OR* odds ratio, *95% CI* 95% confidence interval

*p*- values were calculated by logistic regression adjusted for age and gender

Bold values indicated that the *p*-value was statistically significant

### Association of CYP4V2 SNPs with CHD risk stratified analysis by age and gender

Along with a stratified analysis by age (age > 60 or age ≤ 60) and gender, this study investigated the effect of *CYP4V2* SNPs on CHD risk. As shown in Table 4, rs56413992 was correlated with an increased risk of CHD in the population aged > 60 (heterozygous model: OR = 1.49, 95% CI = 1.02–2.18, *p* = 0.042; dominant model: OR = 1.58, 95% CI = 1.09–2.29, *p* = 0.015; log-additive model: OR = 1.54, 95% CI = 1.12–2.12, *p* = 0.008). In the Table 5, rs56413992 was linked to a higher risk of CHD in males (dominant model: OR = 1.44, 95% CI = 1.04–2.00, *p* = 0.027; log-additive model: OR = 1.43, 95% CI = 1.08–1.90, *p* = 0.013).

**Table 4:** Association of *CYP4V2* gene SNP polymorphisms with CHD risk stratified analysis by age

SNP_ID	Model	Genotypes	Age > 60				Age ≤ 60			
			**Case**	**Control**	**OR (95% CI)**	***p***	**Case**	**Control**	**OR (95% CI)**	***p***
**rs1398007**	Co-dominant	CC	119	128	1		99	112	1	
		CT	124	113	1.12(0.78–1.62)	0.538	98	95	1.18(0.80–1.76)	0.408
		TT	22	21	1.08(0.56–2.10)	0.812	25	18	1.48(0.75–2.91)	0.255
	Dominant	CC	119	128	1		99	112	1	
		CT + TT	146	134	1.12(0.79–1.59)	0.541	123	113	1.23(0.84–1.79)	0.280
	Recessive	CC + CT	243	241	1		197	207	1	
		TT	22	21	1.02(0.54–1.93)	0.944	25	18	1.37(0.71–2.62)	0.344
	Log-additive	—	—	—	1.08(0.82–1.42)	0.608	—	—	1.20(0.90–1.61)	0.211
**rs13146272**	Co-dominant	CC	100	96	1		77	92	1	
		CA	124	121	0.99(0.68–1.46)	0.972	115	104	1.31(0.87–1.96)	0.202
		AA	40	45	0.77(0.46–1.30)	0.323	30	29	1.18(0.65–2.16)	0.580
	Dominant	CC	100	96	1		77	92	1	
		CA + AA	164	166	0.93(0.65–1.33)	0.692	145	133	1.28(0.87–1.89)	0.216
	Recessive	CC + CA	224	217	1		192	196	1	
		AA	40	45	0.77(0.48–1.24)	0.287	30	29	1.02(0.59–1.77)	0.946
	Log-additive	—	—	—	0.90(0.70–1.15)	0.405	—	—	1.14(0.86–1.51)	0.345
**rs3736455**	Co-dominant	TT	93	86	1		75	88	1	
		TG	135	132	0.95(0.65–1.40)	0.972	119	108	1.27(0.84–1.91)	0.259
		GG	37	43	0.73(0.43–1.25)	0.251	28	28	1.13(0.61–2.08)	0.707
	Dominant	TT	93	86	1		75	88	1	
		TG + GG	172	175	0.89(0.62–1.29)	0.546	147	136	1.24(0.84–1.83)	0.288
	Recessive	TT + TG	228	218	1		194	196	1	
		GG	37	43	0.75(0.46–1.23)	0.253	28	28	0.98(0.56–1.73)	0.945
	Log-additive	—	—	—	0.87(0.68–1.13)	0.304	—	—	1.11(0.84–1.48)	0.459
**rs1053094**	Co-dominant	TT	105	120	1		87	89	1	
		TA	128	115	1.24(0.86–1.79)	0.259	105	110	0.93(0.62–1.40)	0.736
		AA	32	27	1.45(0.81–2.60)	0.217	30	25	1.21(0.65–2.25)	0.543
	Dominant	TT	105	120	1		87	89	1	
		TA + AA	160	142	1.28(0.90–1.82)	0.175	135	135	0.98(0.67–1.45)	0.937
	Recessive	TT + TA	233	235	1		192	199	1	
		AA	32	27	1.30(0.74–2.26)	0.360	30	25	1.26(0.71–2.24)	0.437
	Log-additive	—	—	—	1.22(0.93–1.58)	0.148	—	—	1.05(0.79–1.39)	0.747
**rs56413992**	Co-dominant	CC	161	182	1		127	144	1	
		CT	90	74	1.49(1.02–2.18)	**0.042**	85	70	1.33(0.89–1.99)	0.158
		TT	14	6	2.66(0.98–7.21)	0.054	10	9	1.26(0.49–3.24)	0.639
	Dominant	CC	161	182	1		127	144	1	
		CT + TT	104	80	1.58(1.09–2.29)	**0.015**	95	79	1.33(0.90–1.95)	0.153
	Recessive	CC + CT	251	256	1		212	214	1	
		TT	14	6	2.35(0.87–6.30)	0.090	10	9	1.13(0.44–2.88)	0.803
	Log-additive	—	—	—	1.54(1.12–2.12)	**0.008**	—	—	1.25(0.90–1.73)	0.193

*SNP* single nucleotide polymorphism, *OR* odds ratio, *95% CI* 95% confidence interval

*p*- values were calculated by logistic regression adjusted for age and gender

Bold values indicated that the *p*-value was statistically significant

**Table 5:** Association of C*YP4V2* gene SNP polymorphisms with CHD risk stratified analysis by gender

SNP_ID	Model	Genotypes	Male				Female			
			**Case**	**Control**	**OR (95% CI)**	***p***	**Case**	**Control**	**OR (95% CI)**	***p***
**0rs1398007**	Co-dominant	CC	135	159	1		83	81	1	
		CT	155	146	1.25(0.90–1.73)	0.185	67	62	0.92(0.56–1.51)	0.748
		TT	34	24	1.68(0.94–3.00)	0.079	13	15	1.01(0.43–2.37)	0.990
	Dominant	CC	135	159	1		83	81	1	
		CT + TT	189	170	1.31(0.96–1.79)	0.093	80	77	0.94(0.59–1.49)	0.784
	Recessive	CC + CT	290	305	1		150	143	1	
		TT	34	24	1.50(0.86–2.61)	0.150	13	15	1.04(0.45–2.39)	0.924
	Log-additive	—	—	—	1.28(1.00–1.63)	0.052	—	—	0.97(0.68–1.39)	0.864
**rs13146272**	Co-dominant	CC	122	127	1		55	61	1	
		CA	151	155	0.98(0.70–1.37)	0.897	88	70	1.43(0.86–2.38)	0.173
		AA	50	47	1.06(0.66–1.70)	0.822	20	27	0.70(0.34–1.43)	0.323
	Dominant	CC	122	127	1		55	61	1	
		CA + AA	201	202	1.00(0.72–1.37)	0.982	108	97	1.20(0.74–1.95)	0.452
	Recessive	CC + CA	273	282	1		143	131	1	
		AA	50	47	1.07(0.69–1.66)	0.764	20	27	0.57(0.29–1.10)	0.093
	Log-additive	—	—	—	1.02(0.81–1.27)	0.890	—	—	0.94(0.67–1.32)	0.724
**rs3736455**	Co-dominant	TT	119	118	1		49	56	1	
		TG	158	164	0.91(0.65–1.28)	0.596	96	76	1.52(0.90–2.54)	0.116
		GG	47	46	0.97(0.60–1.58)	0.912	18	25	0.73(0.34–1.54)	0.406
	Dominant	TT	119	118	1		49	56	1	
		TG + GG	205	210	0.93(0.67–1.28)	0.639	114	101	1.30(0.79–2.13)	0.296
	Recessive	TT + TG	277	282	1		145	132	1	
		GG	47	46	1.03(0.66–1.60)	0.910	18	25	0.56(0.28–1.12)	0.100
	Log-additive	—	—	—	0.97(0.77–1.22)	0.784	—	—	0.98(0.69–1.39)	0.905
**rs1053094**	Co-dominant	TT	127	141	1		65	68	1	
		TA	153	156	1.09(0.78–1.52)	0.623	80	69	1.12(0.68–1.84)	0.661
		AA	44	32	1.60(0.95–2.70)	0.080	18	20	1.00(0.47–2.13)	0.995
	Dominant	TT	127	141	1		65	68	1	
		TA + AA	197	188	1.17(0.85–1.61)	0.329	98	89	1.09(0.68–1.75)	0.716
	Recessive	TT + TA	280	297	1		145	137	1	
		AA	44	32	1.53(0.93–2.50)	0.092	18	20	0.94(0.46–1.92)	0.869
	Log-additive	—	—	—	1.20(0.95–1.52)	0.124	—	—	1.03(0.73–1.46)	0.851
**rs56413992**	Co-dominant	CC	191	222	1		97	104	1	
		CT	117	98	1.37(0.98–1.91)	0.068	58	46	1.39(0.83–2.30)	0.209
		TT	16	8	2.42(1.00–5.87)	0.051	8	7	1.35(0.45–4.03)	0.596
	Dominant	CC	191	222	1		97	104	1	
		CT + TT	133	106	1.44(1.04–2.00)	**0.027**	66	53	1.38(0.85–2.24)	0.193
	Recessive	CC + CT	308	320	1		155	150	1	
		TT	16	8	2.18(0.90–5.25)	0.083	8	7	1.21(0.41–3.58)	0.730
	Log-additive	—	—	—	1.43(1.08–1.90)	**0.013**	—	—	1.28(0.86–1.91)	0.230

*SNP* single nucleotide polymorphism, *OR* odds ratio, *95% CI* 95% confidence interval

*p*- values were calculated by logistic regression adjusted for age and gender

Bold values indicated that the *p*-value was statistically significant

### Association of CYP4V2 SNPs with CHD risk stratified analysis by smoking and drinking

The effect of *CYP4V2* polymorphisms on CHD risk in subgroups of smoking and drinking was also investigated. As shown in Table 6, the findings suggested that rs56413992 was correlated with a elevated risk of CHD in smokers (dominant model: OR = 1.51, 95% CI = 1.05–2.17, *p* = 0.025; log-additive model: OR = 1.51, 95% CI = 1.09–2.08, *p* = 0.012) and drinkers (heterozygous model: OR = 2.26, 95% CI = 1.45–3.51, *p* < 0.001; dominant model: OR = 2.29, 95% CI = 1.48–3.52, *p* < 0.001; log-additive model: OR = 2.07, 95% CI = 1.40–3.06, *p* < 0.001). In addition, the study also suggested that rs1398007 was significantly linked to an increased risk of CHD in drinkers under homozygous (OR = 1.80, 95% CI = 1.17–2.78, *p* = 0.008), heterozygous (OR = 2.41, 95% CI = 1.11–5.25, *p* = 0.026), dominant (OR = 1.88, 95% CI = 1.24–2.86, *p* = 0.003) and log-additive (OR = 1.65, 95% CI = 1.19–2.30, *p* = 0.003) models, as displayed in the Table 7.

**Table 6:** Association of C*YP4V2* gene SNP polymorphisms with CHD risk stratified analysis by smoking

SNP_ID	Model	Genotypes	Smoking-Yes				Smoking-No			
			**Case**	**Control**	**OR (95% CI)**	***p***	**Case**	**Control**	**OR (95% CI)**	***p***
**rs1398007**	Co-dominant	CC	107	130	1		111	110	1	
		CT	120	109	1.31(0.91–1.90)	0.150	102	99	1.03(0.69–1.53)	0.888
		TT	29	20	1.86(0.98–3.51)	0.056	18	19	0.89(0.43–1.82)	0.748
	Dominant	CC	107	130	1		111	110	1	
		CT + TT	149	129	1.39(0.98–1.98)	0.065	120	118	1.01(0.69–1.47)	0.975
	Recessive	CC + CT	227	239	1		213	209	1	
		TT	29	20	1.63(0.88–2.99)	0.118	18	19	0.88(0.44–1.75)	0.710
	Log-additive	—	—	—	1.34(1.02–1.76)	**0.034**	—	—	0.98(0.73–1.32)	0.891
**rs13146272**	Co-dominant	CC	93	98	1		84	90	1	
		CA	127	127	1.04(0.71–1.52)	0.846	112	98	1.20(0.79–1.82)	0.384
		AA	36	34	1.05(0.60–1.83)	0.857	34	40	0.76(0.43–1.34)	0.348
	Dominant	CC	93	98	1		84	90	1	
		CA + AA	163	161	1.04(0.72–1.50)	0.826	146	138	1.07(0.72–1.58)	0.741
	Recessive	CC + CA	220	225	1		196	188	1	
		AA	36	34	1.03(0.62–1.72)	0.910	34	40	0.69(0.41–1.16)	0.161
	Log-additive	—	—	—	1.03(0.79–1.34)	0.829	—	—	0.93(0.71–1.22)	0.641
**rs3736455**	Co-dominant	TT	91	88	1		77	86	1	
		TG	131	138	0.98(0.55–1.73)	0.941	123	102	1.26(0.83–1.92)	0.270
		GG	34	32	0.91(0.62–1.34)	0.628	31	39	0.76(0.42–1.35)	0.344
	Dominant	TT	91	88	1		77	86	1	
		TG + GG	165	170	0.92(0.64–1.33)	0.669	154	141	1.12(0.75–1.66)	0.577
	Recessive	TT + TG	222	226	1		200	188	1	
		GG	34	32	1.04(0.61–1.75)	0.893	31	39	0.66(0.39–1.12)	0.123
	Log-additive	—	—	—	0.97(0.74–1.27)	0.809	—	—	0.94(0.71–1.24)	0.671
**rs1053094**	Co-dominant	TT	98	114	1		94	95	1	
		TA	127	121	1.21(0.83–1.75)	0.328	106	104	1.00(0.66–1.49)	0.981
		AA	31	23	1.61(0.87–2.97)	0.130	31	29	1.14(0.63–2.06)	0.675
	Dominant	TT	98	114	1		94	95	1	
		TA + AA	158	144	1.27(0.89–1.81)	0.194	137	133	1.03(0.70–1.50)	0.899
	Recessive	TT + TA	225	235	1		200	199	1	
		AA	31	23	1.45(0.81–2.59)	0.207	31	29	1.14(0.65–1.99)	0.647
	Log-additive	—	—	—	1.25(0.95–1.63)	0.115	—	—	1.05(0.79–1.38)	0.750
**rs56413992**	Co-dominant	CC	145	171	1		143	155	1	
		CT	100	83	1.44(0.99–2.08)	0.056	75	61	1.38(0.91–2.10)	0.133
		TT	11	5	2.88(0.96–8.69)	0.060	13	10	1.39(0.58–3.34)	0.465
	Dominant	CC	145	171	1		143	155	1	
		CT + TT	111	88	1.51(1.05–2.17)	**0.025**	88	71	1.38(0.93–2.05)	0.112
	Recessive	CC + CT	245	254	1		218	216	1	
		TT	11	5	2.52(0.84–7.52)	0.098	13	10	1.26(0.53–3.00)	0.607
	Log-additive	—	—	—	1.51(1.09–2.08)	**0.012**	—	—	1.28(0.92–1.78)	0.137

*SNP* single nucleotide polymorphism, *OR* odds ratio, *95% CI* 95% confidence interval

*p*-values were calculated by logistic regression adjusted for age and gender

Bold values indicated that the *p*-value was statistically significant

**Table 7:** Association of C*YP4V2* gene SNP polymorphisms with CHD risk stratified analysis by drinking

SNP_ID	Model	Genotypes	Drinking-Yes				Drinking-No			
			**Case**	**Control**	**OR (95% CI)**	***p***	**Case**	**Control**	**OR (95% CI)**	***p***
**rs1398007**	Co-dominant	CC	67	106	1		151	134	1	
		CT	93	81	1.80(1.17–2.78)	**0.008**	129	127	0.83(0.59–1.18)	0.301
		TT	20	13	2.41(1.11–5.25)	**0.026**	27	26	0.89(0.49–1.61)	0.698
	Dominant	CC	67	106	1		151	134	1	
		CT + TT	113	94	1.88(1.24–2.86)	**0.003**	156	153	0.84(0.61–1.17)	0.309
	Recessive	CC + CT	160	187	1		280	261	1	
		TT	20	13	1.79(0.85–3.77)	0.124	27	26	0.97(0.55–1.72)	0.914
	Log-additive	—	—	—	1.65(1.19–2.30)	**0.003**	—	—	0.90(0.70–1.16)	0.404
**rs13146272**	Co-dominant	CC	76	73	1		101	115	1	
		CA	86	98	0.83(0.53–1.29)	0.400	153	127	1.46(1.01–2.09)	**0.042**
		AA	18	29	0.58(0.29–1.14)	0.116	52	45	1.26(0.77–2.05)	0.354
	Dominant	CC	76	73	1		101	115	1	
		CA + AA	104	127	0.77(0.51–1.17)	0.224	205	172	1.40(1.00–1.97)	0.052
	Recessive	CC + CA	162	171	1		254	242	1	
		AA	18	29	0.64(0.34–1.21)	0.174	52	45	1.02(0.66–1.59)	0.920
	Log-additive	—	—	—	0.78(0.57–1.07)	0.118	—	—	1.18(0.93–1.49)	0.163
**rs3736455**	Co-dominant	TT	74	69	1		94	105	1	
		TG	89	101	0.81(0.52–1.25)	0.337	165	139	1.41(0.98–2.02)	0.066
		GG	17	29	0.54(0.27–1.07)	0.078	48	42	1.23(0.74–2.04)	0.425
	Dominant	TT	74	69	1		94	105	1	
		TG + GG	106	130	0.75(0.49–1.14)	0.174	213	181	1.36(0.96–1.93)	0.080
	Recessive	TT + TG	163	170	1		259	244	1	
		GG	17	29	0.61(0.32–1.16)	0.130	48	42	1.00(0.64–1.58)	0.990
	Log-additive	—	—	—	0.75(0.55–1.03)	0.079	—	—	1.17(0.91–1.49)	0.216
**rs1053094**	Co-dominant	TT	65	88	1		127	121	1	
		TA	92	95	1.24(0.80–1.93)	0.332	141	130	1.01(0.71–1.42)	0.977
		AA	23	17	2.04(0.99–4.20)	0.055	39	35	1.11(0.66–1.88)	0.701
	Dominant	TT	65	88	1		127	121	1	
		TA + AA	115	112	1.36(0.89–2.06)	0.156	180	165	1.03(0.74–1.43)	0.877
	Recessive	TT + TA	157	183	1		268	251	1	
		AA	23	17	1.81(0.91–3.60)	0.090	39	35	1.11(0.67–1.81)	0.690
	Log-additive	—	—	—	1.36(0.99–1.87)	0.061	—	—	1.04(0.82–1.32)	0.758
**rs56413992**	Co-dominant	CC	94	143	1		194	183	1	
		CT	80	53	2.26(1.45–3.51)	** < 0.001**	95	91	1.04(0.73–1.48)	0.846
		TT	6	4	2.66(0.70–10.10)	0.150	18	11	1.59(0.73–3.49)	0.245
	Dominant	CC	94	143	1		194	183	1	
		CT + TT	86	57	2.29(1.48–3.52)	** < 0.001**	113	102	1.10(0.78–1.54)	0.593
	Recessive	CC + CT	174	196	1		289	274	1	
		TT	6	4	2.00(0.53–7.52)	0.305	18	11	1.57(0.72–3.42)	0.252
	Log-additive	—	—	—	2.07(1.40–3.06)	** < 0.001**	—	—	1.13(0.86–1.50)	0.386

*SNP* single nucleotide polymorphism, *OR* odds ratio, *95% CI* 95% confidence interval

*p*-values were calculated by logistic regression adjusted for age and gender

Bold values indicated that the *p*-value was statistically significant

### The effect of *CYP4V2* SNPs on the risk of CHD complicated with DM or HTN

In this study, we also selected CHD patients with DM or HTN as the case group, and CHD patients without DM or HTN as the control group, respectively, to explore the association of *CYP4V2* SNPs with the risk of CHD complicated with or without DM or HTN. As shown in Table 8, rs1053094 was associated with a reduced risk of CHD complicated with DM compared with CHD patients without DM (homozygous model: OR = 0.63, 95% CI = 0.42–0.96, *p* = 0.032; heterozygous model: OR = 0.48, 95% CI = 0.24–0.94, *p* = 0.033; dominant model: OR = 0.60, 95% CI = 0.40–0.89, *p* = 0.011; log-additive model: OR = 0.67, 95% CI = 0.49–0.91, *p* = 0.010). At the same time, rs1398007 was related to a decrease risk of CHD complicated with HTN compared with CHD patients without HTN (heterozygous model: OR = 0.57, 95% CI = 0.38–0.84, *p* = 0.005; dominant model: OR = 0.61, 95% CI = 0.42–0.90, *p* = 0.012).

**Table 8:** The effect of CYP4V2 gene polymorphisms on whether CHD was complicated by diabetes mellitus (DM) or hypertension (HTN) risk

SNP_ID	Model	Genotypes	Case (With DM)	Controls (Without DM)	OR (95% CI)	*p*	Case (With HTN)	Controls (Without HTN)	OR (95% CI)	*p*
**rs1398007**	Co-dominant	CC	62	156	1		150	68	1	
		CT	63	159	1.03(0.67–1.57)	0.908	122	100	0.57(0.38–0.84)	**0.005**
		TT	18	29	1.64(0.894–3.21)	0.147	31	16	0.90(0.46–1.78)	0.764
	Dominant	CC	62	156	1		150	68	1	
		CT + TT	81	188	1.12(0.75–1.67)	0.586	153	116	0.61(0.42–0.90)	**0.012**
	Recessive	CC + CT	125	315	1		272	168	1	
		TT	18	29	1.62(0.86–3.05)	0.135	31	16	1.22(0.64–2.33)	0.524
	Log-additive	—	—	—	1.19(0.88–1.61)	0.269	—	—	0.79(0.59–1.05)	0.102
**rs13146272**	Co-dominant	CC	59	118	1		111	66	1	
		CA	59	180	0.65(0.42–1.01)	0.057	149	90	0.96(0.64–1.46)	0.862
		AA	25	45	1.05(0.58–1.90)	0.870	43	21	0.87(0.49–1.57)	0.651
	Dominant	CC	59	118	1		111	66	1	
		CA + AA	84	225	0.74(0.49–1.11)	0.140	192	117	0.94(0.64–1.40)	0.768
	Recessive	CC + CA	118	298	1		260	156	1	
		AA	25	45	1.33(0.77–2.28)	0.304	43	27	0.89(0.52–1.52)	0.676
	Log-additive	—	—	—	0.93(0.69–1.24)	0.612	—	—	0.94(0.71–1.24)	0.670
**rs3736455**	Co-dominant	TT	56	112	1		106	62	1	
		TG	62	192	0.63(0.41–0.97)	**0.037**	155	99	0.87(0.57–1.31)	0.494
		GG	25	40	1.18(0.64–2.16)	0.593	42	23	0.98(0.53–1.81)	0.956
	Dominant	TT	56	112	1		106	62	1	
		TG + GG	87	232	0.72(0.48–1.10)	0.126	197	122	0.89(0.60–1.32)	0.558
	Recessive	TT + TG	118	304	1		261	161	1	
		GG	25	40	1.55(0.89–2.68)	0.122	42	23	1.07(0.61–1.87)	0.805
	Log-additive	—	—	—	0.96(0.71–1.29)	0.771	—	—	0.96(0.72–1.27)	0.768
**rs1053094**	Co-dominant	TT	69	123	1		122	70	1	
		TA	61	172	0.63(0.42–0.96)	**0.032**	144	89	0.92(0.61–1.38)	0.687
		AA	13	49	0.48(0.24–0.94)	**0.033**	37	25	0.88(0.49–1.60)	0.685
	Dominant	TT	69	123	1		122	70	1	
		TA + AA	74	221	0.60(0.40–0.89)	**0.011**	181	144	0.91(0.62–1.34)	0.640
	Recessive	TT + TA	130	295	1		266	159	1	
		AA	13	49	0.60(0.32–1.16)	0.129	27	25	0.92(0.53–1.61)	0.782
	Log-additive	—	—	—	0.67(0.49–0.91)	**0.010**	—	—	0.93(0.71–1.23)	0.633
**rs56413992**	Co-dominant	CC	90	129	1		180	108	1	
		CT	46	17	0.83(0.54–1.27)	0.386	108	67	1.02(0.69–1.52)	0.922
		TT	7	90	0.86(0.34–2.17)	0.755	15	9	0.95(0.40–2.29)	0.911
	Dominant	CC	90	198	1		180	108	1	
		CT + TT	53	146	0.83(0.55–1.25)	0.378	123	76	1.01(0.69–1.48)	0.953
	Recessive	CC + CT	136	327	1		288	175	1	
		TT	7	14	0.92(0.37–2.29)	0.861	15	9	0.94(0.40–2.25)	0.897
	Log-additive	—	—	—	0.87(0.62–1.22)	0.427	—	—	1.00(0.73–1.38)	0.999

*SNP* single nucleotide polymorphism, *DM* diabetes mellitus, *HTN* hypertension, *OR* odds ratio, *95% CI* 95% confidence interval

*p*- values were calculated by logistic regression adjusted for age and gender

Bold values indicated that the *p*-value was statistically significant

### Correlation between *CYP4V2* polymorphisms and serum lipid levels

Additionally, we also investigated the relationship between *CYP4V2* polymorphisms and serum lipid levels (TC, TG, HDL-C and LDL-C) in CHD patients (Table 9). The results revealed that five candidate SNPs were not correlated with the levels of TC, TG, HDL-C and LDL-C in the CHD patients.

**Table 9:** Association of *CYP4V2* gene polymorphisms with serum lipid levels

SNP_ID	Genotype	TC (mmol/L)		TG (mmol/L)		HDL-C (mmol/L)		LDL-C (mmol/L)	
		**n**	**Mean ± SD**	**n**	**Mean ± SD**	**n**	**Mean ± SD**	**n**	**Mean ± SD**
**rs1398007**	CC	207	4.11 ± 1.01	207	1.69 ± 1.11	206	1.13 ± 0.27	205	2.47 ± 0.84
	CT	210	4.04 ± 1.08	210	1.58 ± 0.90	209	1.10 ± 0.27	209	2.34 ± 0.89
	TT	45	4.07 ± 1.02	45	1.53 ± 1.02	45	1.10 ± 0.20	45	2.64 ± 1.42
	*p*		0.754		0.419		0.589		0.109
**rs13146272**	CC	168	4.08 ± 1.03	168	1.59 ± 0.94	168	1.12 ± 0.29	168	2.44 ± 1.02
	CA	228	4.09 ± 1.05	228	1.70 ± 1.14	226	1.11 ± 0.24	225	2.42 ± 0.88
	AA	65	4.02 ± 1.05	65	1.43 ± 0.62	65	1.11 ± 0.27	65	2.44 ± 0.94
	*p*		0.900		0.125		0.842		0.956
**rs3736455**	TT	160	4.03 ± 1.02	160	1.58 ± 0.96	160	1.12 ± 0.30	160	2.41 ± 1.04
	TG	242	4.11 ± 1.07	242	1.71 ± 1.11	240	1.11 ± 0.24	239	2.44 ± 0.88
	GG	60	4.03 ± 1.00	60	1.40 ± 0.63	60	1.12 ± 0.27	60	2.45 ± 0.91
	*p*		0.736		0.082		0.939		0.928
**rs1053094**	TT	183	4.11 ± 1.09	183	1.68 ± 1.16	182	1.12 ± 0.26	182	2.46 ± 0.87
	TA	219	4.06 ± 1.02	219	1.60 ± 0.96	218	1.12 ± 0.28	218	2.41 ± 0.99
	AA	60	4.03 ± 0.98	60	1.51 ± 0.62	60	1.09 ± 0.22	59	2.42 ± 0.92
	*p*		0.858		0.482		0.692		0.849
**rs56413992**	CC	275	4.04 ± 1.07	275	1.60 ± 1.06	274	1.12 ± 0.27	274	2.41 ± 0.89
	CT	164	4.16 ± 0.99	164	1.68 ± 0.97	163	1.12 ± 0.25	162	2.47 ± 1.02
	TT	23	3.92 ± 0.99	23	1.44 ± 0.46	23	1.07 ± 0.23	23	2.38 ± 0.95
	*p*		0.399		0.480		0.762		0.827

*SNP* single nucleotide polymorphism, *TC* total cholesterol, *TG* triglyceride, *HDL-C* high-density lipoprotein cholesterol, *LDL-C* low-density lipoprotein cholesterol, *n* number, *SD* standard deviation

*p*-values were calculated using *χ*^2^ tests

### LD and haplotype analysis

**Fig. 3:**
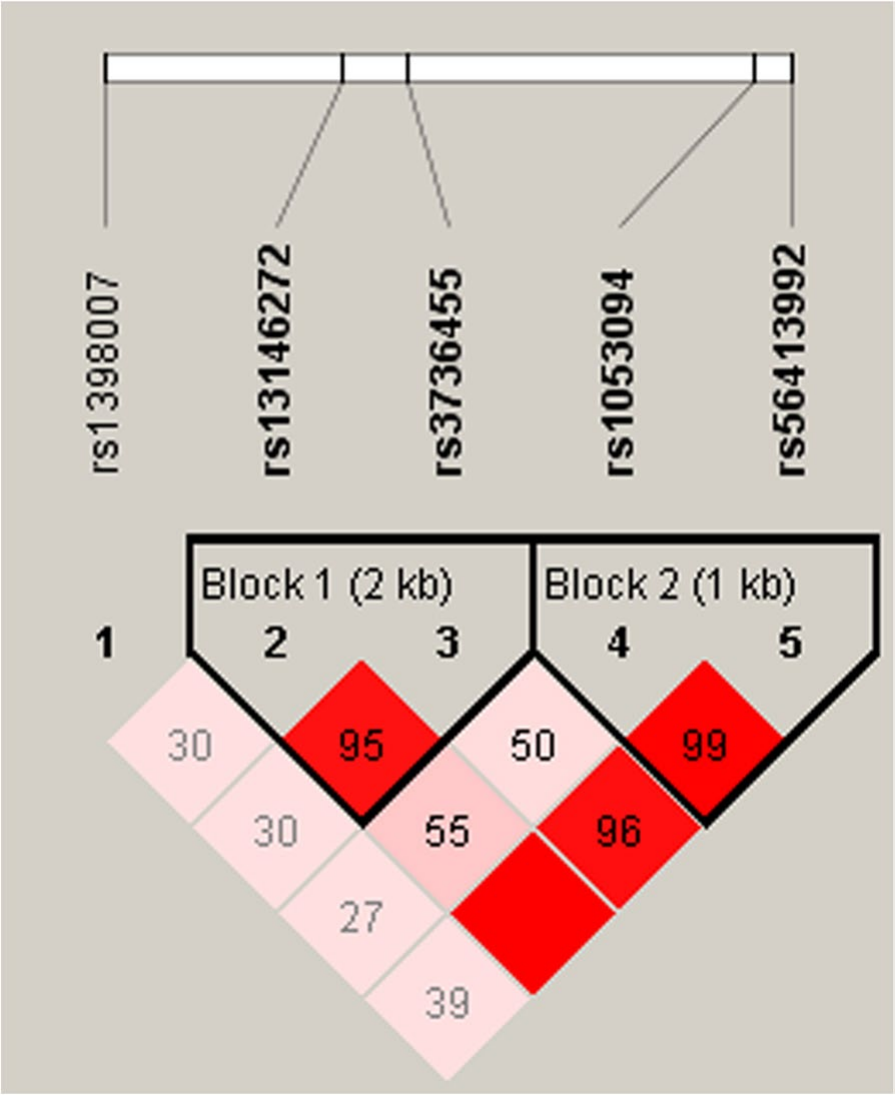
Haplotype block map of *CYP4V2* variants. The numbers inside the diamonds indicate the D′for pairwise analyses

We performed the LD analysis among five variants (rs1398007, rs13146272, rs3736455, rs1053094 and rs56413992), as shown in Fig. 3. Rs13146272 and rs3736455 formed 2kb-block 1. Rs1053094 and rs56413992 formed 1kb-block 2. But, no haplotype was found to be associated with CHD risk.

### MDR analysis

**Fig. 4:**
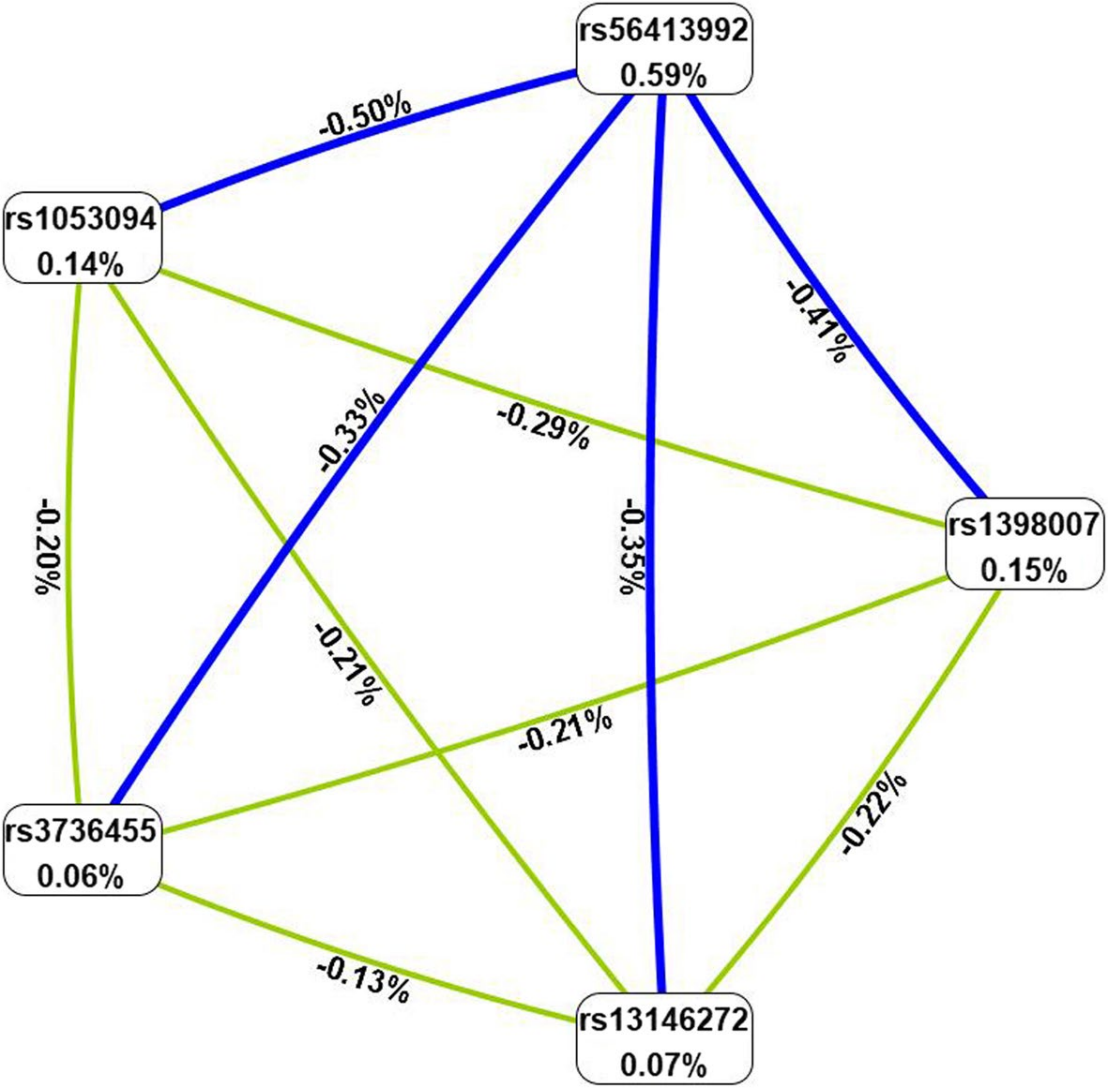
Circle graph of the effect of SNP-SNP interaction on CHD risk. A negative value of the two-locus entropy indicates an antagonistic effect, while a positive value indicates a synergistic effect

MDR analysis was used to predict the impact of SNP-SNP interaction on CHD risk. As shown in Table 10, the best model for predicting CHD risk was the five loci model (OR = 1.84, 95% CI = 1.15–2.94, *p* = 0.010) with cross-validation 10/10 and a balance test accuracy of 0.486. The circle graph (Fig. 4) showed that interaction among five SNPs (rs1398007, rs13146272, rs3736455, rs1053094 and rs56413992) had a strong antagonistic effect on *CYP4V2* gene.

**Table 10:** Multifactor dimensionality reduction (MDR) analysis

Model	Training Bal. Acc	Testing Bal. Acc	CVC	OR (95% CI)	*p*
rs56413992	0.528	0.504	6/10	1.44(1.1–1.87)	**0.007**
rs1053094, rs56413992	0.529	0.514	7/10	1.33(1.02–1.72)	**0.035**
rs1398007, rs3736455, rs56413992	0.526	0.495	7/10	1.47(1.04–2.09)	**0.030**
rs1398007, rs3736455, rs1053094, rs56413992	0.529	0.504	5/10	1.89(1.19–2.99)	**0.006**
rs1398007, rs13146272, rs3736455, rs1053094, rs56413992	0.523	0.486	10/10	1.84(1.15–2.94)	**0.010**

*MDR* multi-factor dimensionality reduction, *Bal. Acc.* balanced accuracy, *CVC* cross-validation consistency, *OR* odds ratio, *95% CI* 95% confidence interval

Bold values indicated that the *p*-value was statistically significant

*p*-values were calculated using *χ*^2^ tests

### FPRP analysis

In the FPRP analysis, the FPRP values under different prior probabilities were observed, and FPRP values < 0.2 were considered to indicate significant correlation (Supplementary table 2). When the prior probability was 0.25, all statistically significant findings were notable except that rs1398007 TT genotype increased the risk of CHD in drinkers and rs1053094 AA genotype increased the risk of CHD complicated with DM population. When the prior probability was 0.1, the significant association of rs56413992 with an increased risk of CHD in the overall analysis was more noteworthy (T vs C model: FPRP = 0.072, statistical power = 0.805; CT vs CC model: FPRP = 0.152, statistical power = 0.693; CT + TT vs CC model: FPRP = 0.113, statistical power = 0.637; Log-additive model: FPRP = 0.071, statistical power = 0.783).

## Discussion

CHD is a complex polygenic genetic disease affected by genetic factors. At present, several gene SNPs have been found to be associated with CHD risk, such as *UTS2* (Ser89Asn) [18], *CDKN2B-AS1* (rs10738606) [1], *GLUT4* (rs5418) [19], *CYP11B1* (rs4534, rs6410 and rs5283) [20], *CYP24A1* (rs6068816 and rs2296241) [21] and *AGT* (rs2493132) [22]. Considering that the connection of *CYP4V2* polymorphisms with CHD risk has not been reported, five SNPs (rs1398007, rs13146272, rs3736455, rs1053094 and rs56413992) in *CYP4V2* gene were eventually genotyped in this study and their association with CHD risk were explored. As a result, this study suggested that *CYP4V2* rs56413992 T allele and CT genotype significantly increased the risk of CHD. So *CYP4V2*-rs56413992 might play a critical part in the pathogenesis of CHD.

*CYP4V2* is often referred to as an orphan P450 because its substrate specificity is just beginning to be defined [11]. It has been reported that *CYP4V2* gene is involved in the metabolism of lipids (endogenous fatty acids and steroids) by ω-hydroxylation [14]. Interestingly, lipid metabolism also plays a chief part in the pathogenesis of CHD. Recent studies have suggested that atherosclerosis is a key pathological basis of CHD, and one of its main features is lipid deposition caused by abnormal lipid metabolism in the arterial intima [23–25]. Therefore, we speculated that *CYP4V2* was involved in the pathogenesis of CHD by participating in lipid metabolism, and this study was devoted to investigating the impact of *CYP4V2* SNPs on CHD risk.

Multiple studies on *CYP4V2* polymorphisms have revealed the relationship between rs13146272 and venous thrombosis [26–29]. For example, a recent study has found that *CYP4V2*-rs13146272 is closely linked to the occurrence of venous thromboembolism (VTE) [15]. However, no connection of rs13146272 with CHD risk was observed in our study, which may be related to differences in disease. By contrast, little is known about the SNPs (rs1398007, rs3736455, rs1053094 and rs56413992) in *CYP4V2*. Therefore, our study is the first to report the association of these SNPs with CHD risk and find that rs56413992, as a risk factor, may play a crucial role in the pathogenesis of CHD.

There are age and gender differences in the occurrence of CHD. According to statistics, the incidence of CHD in people under the age of 40 is lower (0.6%), but with the gradual increase of age, the incidence of CHD will continue to increase [30]. In an age-based stratified analysis, we found that rs56413992 was significantly related to a greater risk of CHD in individuals aged > 60 years, suggesting that rs56413992 was a risk factor for CHD in individuals aged > 60 years. This further confirmed the previous results. In addition, one study has found that CHD is more common in men than women [31, 32]. In our study, we found that rs56413992 significantly increased CHD risk in men, but not in women. Based on this, we speculated that rs56413992 played a great part in the development of CHD in men, which may be closely related to men’s living habits, such as smoking and drinking.

Current researches have shown that smoking and drinking are the main risk factors for CHD. Active smoking and second-hand smoke exposure are estimated to cause more than 30% of CHD mortality, and smokers have twice the risk of fatal event within 10 years compared to non-smokers [33]. Furthermore, numerous epidemiological academic researches have suggested a J-shaped linkage between drinking and CHD morbidity and mortality, that is, non-drinkers and heavy drinkers have a slightly higher risk of CHD compared with light drinkers [34, 35]. This may have a strong relationship with moderate drinking to promote blood circulation, and further experiments are needed to confirm it. Our discovery indicated that rs1398007 increased the risk of CHD in drinkers, and rs56413992 increased CHD risk in smokers and drinkers, suggesting that *CYP4V2* rs1398007 and rss56413992 may be a higher risk factor for CHD patients with smoking or alcohol consumption.

In addition, CHD complications HNT and DM are believed to play an integral role in the progression of CHD. Studies have shown that DM is a traditional risk factor for CHD, which exacerbates the progression of atherosclerosis and leads to poor clinical outcomes [36, 37]. Studies have also shown that hypertension accounts for more than 45% of all CHD events worldwide and it is an important risk factor for CHD [38]. In our study, we explored the association of *CYP4V2* SNPs with CHD complications (HTN and DM) in the CHD group. The results implied that rs1053094 was related to a lower risk of CHD complicated with DM, and rs1398007 was correlated with a decreased risk of CHD complicated with HTN. However, no significant association of rs1053094 and rs1398007 with CHD risk was observed in the overall analysis. Therefore, this result needs to be validated with a larger sample.

Although our study explored the relationship between *CYP4V2* rs56413992 and CHD risk, and suggested that rs56413992 may be a risk factor for CHD, this study also has some limitations. It is well known that there is a strong linkage between abnormal lipid metabolism and an increased risk of CHD. However, the association between blood lipid influencing factors and CHD risk were not explored in our study. In the follow-up study, we will fully consider and incorporate corresponding indicators affecting blood lipids to further study the relationship between *CYP4V2* polymorphisms and CHD risk.

## Conclusions

This study revealed a potential association between *CYP4V2* rs56413992 polymorphisms and the risk and progression of CHD. This study will provide new clues for the early prevention, diagnosis and treatment of CHD.

### Supplementary Information

**Additional file 1: Supplementary Table 1.** The primers of the selected SNPs. **Supplementary Table 1.** The primers of the selected SNPs.

## Data Availability

The data that support the findings of this study are available from the corresponding author upon reasonable request.

## References

[1] Huang K, Zhong J, Li Q, Zhang W, Chen Z, Zhou Y, Wu M, Zhong Z, Lu S, Zhang S (2019). Effects of CDKN2B-AS1 polymorphisms on the susceptibility to coronary heart disease. Mol Genet Genomic Med.

[2] Charla E, Mercer J, Maffia P, Nicklin SA (2020). Extracellular vesicle signalling in atherosclerosis. Cell Signal.

[3] Ghafouri-Fard S, Gholipour M, Taheri M (2021). The emerging role of long non-coding RNAs and circular RNAs in coronary artery disease. Front Cardiovasc Med.

[4] Xia Y, Brewer A, Bell JT (2021). DNA methylation signatures of incident coronary heart disease: findings from epigenome-wide association studies. Clin Epigenetics.

[5] Hua L, Yuan JX, He S, Zhao CH, Jia QW, Zhang J, An FH, Chen ZH, Li LH, Wang LS (2020). Analysis on the polymorphisms of site RS4977574, and RS1333045 in region 9p21 and the susceptibility of coronary heart disease in Chinese population. BMC Med Genet.

[6] Lu WH, Zhang WQ, Zhao YJ, Gao YT, Tao N, Ma YT, Liu JW, Wulasihan M (2020). Case-control study on the interaction effects of rs10757278 polymorphisms at 9p21 locus and traditional risk factors on coronary heart disease in Xinjiang. China J Cardiovasc Pharmacol.

[7] Drenos F, Grossi E, Buscema M, Humphries SE (2015). Networks in coronary heart disease genetics as a step towards systems epidemiology. PLoS ONE.

[8] Mega JL, Stitziel NO, Smith JG, Chasman DI, Caulfield M, Devlin JJ, Nordio F, Hyde C, Cannon CP, Sacks F (2015). Genetic risk, coronary heart disease events, and the clinical benefit of statin therapy: an analysis of primary and secondary prevention trials. Lancet.

[9] Pranavchand R, Reddy BM (2013). Current status of understanding of the genetic etiology of coronary heart disease. J Postgrad Med.

[10] Miller CL, Assimes TL, Montgomery SB, Quertermous T (2014). Dissecting the causal genetic mechanisms of coronary heart disease. Curr Atheroscler Rep.

[11] Kelly E, Nakano M, Rohatgi P, Yarov-Yarovoy V, Rettie A (2011). Finding homes for orphan cytochrome P450s: CYP4V2 and CYP4F22 in disease states. Mol Interventions.

[12] Zhang X, Xu K, Dong B, Peng X, Li Q, Jiang F, Xie Y, Tian L, Li Y (2018). Comprehensive screening of CYP4V2 in a cohort of Chinese patients with Bietti crystalline dystrophy. Mol Vis.

[13] Mackay D, Halford S (2012). Focus on molecules: cytochrome P450 family 4, subfamily V, polypeptide 2 (CYP4V2). Exp Eye Res.

[14] Nakano M, Kelly EJ, Rettie AE (2009). Expression and characterization of CYP4V2 as a fatty acid omega-hydroxylase. Drug Metab Dispos.

[15] Yue Y, Sun Q, Man C, Fu Y (2019). Association of the CYP4V2 polymorphism rs13146272 with venous thromboembolism in a Chinese population. Clin Exp Med.

[16] Long F, Wang D, Su Q, Zhang Y, Li J, Xia S, Wang H, Wu Y, Qu Q (2022). CYP4 subfamily V member 2 (CYP4V2) polymorphisms were associated with ischemic stroke in Chinese Han population. BMC Med Genomics.

[17] Rovite V, Maurins U, Megnis K, Vaivade I, Pečulis R, Rits J, Prave S, Klovins J (2014). Association of F11 polymorphism rs2289252 with deep vein thrombosis and related phenotypes in population of Latvia. Thromb Res.

[18] Zhao J, Gu HP, Jiang J, Jie W, Lin L, Han XN, Chu SY, Xue L, Ding WH (2018). Genetic polymorphisms of UTS2 rs2890565 Ser89Asn in coronary heart disease and myocardial infarction in Chinese population. Int J Clin Exp Pathol.

[19] Yu F, Liu F, Li XM, Zhao Q, Luo JY, Zhang JY, Yang YN (2022). GLUT4 gene rs5418 polymorphism is associated with increased coronary heart disease risk in a Uygur Chinese population. BMC Cardiovasc Disord.

[20] Huang X, Cheng Y, Wang N (2022). Genetic variants in CYP11B1 influence the susceptibility to coronary heart disease. BMC Med Genomics.

[21] Qian P, Cao X, Xu X, Duan M, Zhang Q, Huang G (2020). Contribution of CYP24A1 variants in coronary heart disease among the Chinese population. Lipids Health Dis.

[22] Dong M, Liu S, Wang M, Wang Y, Xin Y, Xuan S (2021). Relationship between AGT rs2493132 polymorphism and the risk of coronary artery disease in patients with NAFLD in the Chinese Han population. J Int Med Res.

[23] Fan X, Li A, Yan Z, Geng X, Lian L, Lv H, Gao D, Zhang J (2022). From iron metabolism to ferroptosis: pathologic changes in coronary heart disease. Oxid Med Cell Longev.

[24] Jebari-Benslaiman S, Galicia-García U, Larrea-Sebal A, Olaetxea JR, Alloza I, Vandenbroeck K, Benito-Vicente A, Martín C. Pathophysiology of Atherosclerosis. Int J Mol Sci. 2022;23(6):3346. 10.3390/ijms23063346 PMC8954705 35328769

[25] Björkegren JLM, Lusis AJ (2022). Atherosclerosis: recent developments. Cell.

[26] Sun NA, Cheng P, Deng DH, Liu RR, Lai YR (2016). Analysis of the genetic variants associated with recurrent thromboembolism in a patient with hemoglobin H disease following splenectomy: a case report. Biomed Rep.

[27] Bezemer ID, Bare LA, Doggen CJ, Arellano AR, Tong C, Rowland CM, Catanese J, Young BA, Reitsma PH, Devlin JJ (2008). Gene variants associated with deep vein thrombosis. JAMA.

[28] Austin H, De Staercke C, Lally C, Bezemer ID, Rosendaal FR, Hooper WC (2011). New gene variants associated with venous thrombosis: a replication study in white and black Americans. J Thromb Haemost.

[29] Jiang J, Liu K, Zou J, Ma H, Yang H, Zhang X, Jiao Y (2017). Associations between polymorphisms in coagulation-related genes and venous thromboembolism: a meta-analysis with trial sequential analysis. Medicine (Baltimore).

[30] Yan YL, Qiu B, Hu LJ, Jing XD, Liu YJ, Deng SB, Du JL, She Q (2013). Efficacy and safety evaluation of intensive statin therapy in older patients with coronary heart disease: a systematic review and meta-analysis. Eur J Clin Pharmacol.

[31] Feitosa MF, Kuipers AL, Wojczynski MK, Wang L, Barinas-Mitchell E, Kulminski AM, Thyagarajan B, Lee JH, Perls T, Christensen K (2021). Heterogeneity of the predictive polygenic risk scores for coronary heart disease age-at-onset in three different coronary heart disease family-based ascertainments. Circ Genom Precis Med.

[32] Minhas A, Cubero Salazar I, Kazzi B, Hays AG, Choi AD, Arbab-Zadeh A, Michos ED (2021). Sex-specific plaque signature: uniqueness of atherosclerosis in women. Curr Cardiol Rep.

[33] Gallucci G, Tartarone A, Lerose R, Lalinga AV, Capobianco AM (2020). Cardiovascular risk of smoking and benefits of smoking cessation. J Thorac Dis.

[34] Ikehara S, Iso H, Toyoshima H, Date C, Yamamoto A, Kikuchi S, Kondo T, Watanabe Y, Koizumi A, Wada Y (2008). Alcohol consumption and mortality from stroke and coronary heart disease among Japanese men and women: the Japan collaborative cohort study. Stroke.

[35] Wood AM, Kaptoge S, Butterworth AS, Willeit P, Warnakula S, Bolton T, Paige E, Paul DS, Sweeting M, Burgess S (2018). Risk thresholds for alcohol consumption: combined analysis of individual-participant data for 599 912 current drinkers in 83 prospective studies. Lancet.

[36] Liu J, Zou Y, Tang Y, Xi M, Xie L, Zhang Q, Gong J (2016). Circulating cell-free mitochondrial deoxyribonucleic acid is increased in coronary heart disease patients with diabetes mellitus. J Diabetes Investig.

[37] Yang T, Liu Y, Li L, Zheng Y, Wang Y, Su J, Yang R, Luo M, Yu C (2022). Correlation between the triglyceride-to-high-density lipoprotein cholesterol ratio and other unconventional lipid parameters with the risk of prediabetes and type 2 diabetes in patients with coronary heart disease: a RCSCD-TCM study in China. Cardiovasc Diabetol.

[38] Lukács Krogager M, Skals RK, Appel EVR, Schnurr TM, Engelbrechtsen L, Have CT, Pedersen O, Engstrøm T, Roden DM, Gislason G (2018). Hypertension genetic risk score is associated with burden of coronary heart disease among patients referred for coronary angiography. PLoS ONE.

