# Supplementary material for: CYP4V2 rs56413992 C > T was associated with the risk of coronary heart disease in the Chinese Han population: a case–control study
Source: BMC Med Genomics. 2023 Dec 8;16:322. doi: 10.1186/s12920-023-01737-y (PMC10709878; doi:10.1186/s12920-023-01737-y)
Supplement: Supplementary file 1 — Additional file 1: Supplementary Table 1. The primers of the selected SNPs. Supplementary Table 1. The primers of the selected SNPs. [file 12920_2023_1737_MOESM1_ESM.docx]

| **SNP_ID** | **1st-PCRP** | **2nd-PCRP** | **UEP_SEQ** | **Director** |
| --- | --- | --- | --- | --- |
| **rs1398007** | ACGTTGGATGGCGCAGGAACAGCCCCGTG | ACGTTGGATGACTTCCCCGCGCGGACCTG | ggCCCGGTCCCCGGAAC | R |
| **rs13146272** | ACGTTGGATGTGGCTTTGGCTTGATCTCTG | ACGTTGGATGTCAGGGACTTACACTGTTGG | TGGTAAAAGTATGTAGGATCT | R |
| **rs3736455** | ACGTTGGATGCTGCGTTTATTTTTGGAGGG | ACGTTGGATGAGTCCAAACAGAAGCATGTG | TCAAATTATACAGGTCATCGC | F |
| **rs1053094** | ACGTTGGATGCAGATTTTATAACCTGGAGC | ACGTTGGATGGTGACTGGGATTCTATGAGG | ggtAAGACTATAAGTGCACGA | R |
| **rs56413992** | ACGTTGGATGGAAAAAGTCCTTCCAAGATG | ACGTTGGATGCATGTAGGGTCACTAAAAAC | AAAACAAAATGTAAGGGATAAAT | R |

**Supplementary table 1.** **The primers of the selected SNPs.**

SNP: single nucleotide polymorphism; PCRP: polymerase chain reaction primer; UEP: unextended primer; SEQ: sequence.

**Supplementary table 2.** **Results of FPRP analysis for significant findings.**

| **SNP** | **OR (95% CI)** | ***p*** | **Statistical power** | **Prior probability** | | | | |
| --- | --- | --- | --- | --- | --- | --- | --- | --- |
|  |  |  |  | **0.25** | **0.1** | **0.01** | **0.001** | **0.0001** |
| **rs1398007 (C > T)** |  |  |  |  |  |  |  |  |
| **Smoking-Yes** |  |  |  |  |  |  |  |  |
| Log-additive | 1.34(1.02-1.76) | **0.035** | **0.791** | **0.118** | 0.287 | 0.816 | 0.978 | 0.998 |
| **Drinking-Yes** |  |  |  |  |  |  |  |  |
| CT vs CC | 1.80(1.17-2.78) | **0.008** | **0.206** | **0.105** | 0.260 | 0.795 | 0.975 | 0.997 |
| TT vs CC | 2.41(1.11-5.25) | **0.027** | **0.116** | 0.409 | 0.675 | 0.958 | 0.996 | 1.000 |
| CT+TT vs CC | 1.88(1.24-2.86) | **0.003** | **0.146** | **0.062** | **0.164** | 0.684 | 0.956 | 0.995 |
| Log-additive | 1.65(1.19-2.30) | **0.003** | **0.287** | **0.032** | **0.089** | 0.519 | 0.916 | 0.991 |
| **CHD-With HTN vs CHD-Without HTN** |  |  |  |  |  |  |  |  |
| CT vs CC | 0.57(0.38-0.84) | **0.004** | **0.214** | **0.059** | **0.159** | 0.675 | 0.954 | 0.995 |
| CT+TT vs CC | 0.61(0.42-0.90) | **0.013** | **0.327** | **0.105** | 0.259 | 0.794 | 0.975 | 0.997 |
| **rs1053094 (T > A)** |  |  |  |  |  |  |  |  |
| **CHD-With DM vs CHD-Without DM** |  |  |  |  |  |  |  |  |
| TA vs TT | 0.63(0.42-0.96) | **0.032** | **0.396** | **0.193** | 0.418 | 0.887 | 0.988 | 0.999 |
| AA vs TT | 0.48(0.24-0.94) | **0.032** | **0.169** | 0.365 | 0.632 | 0.950 | 0.995 | 0.999 |
| TA+AA vs TT | 0.60(0.40-0.89) | **0.011** | **0.300** | **0.100** | 0.250 | 0.786 | 0.974 | 0.997 |
| Log-additive | 0.67(0.49-0.91) | **0.010** | **0.513** | **0.057** | **0.154** | 0.667 | 0.953 | 0.995 |
| **rs56413992 (C > T)** |  |  |  |  |  |  |  |  |
| **Overall analysis** |  |  |  |  |  |  |  |  |
| T vs C | 1.36(1.09-1.70) | **0.007** | 0.805 | **0.025** | **0.072** | 0.460 | 0.896 | 0.988 |
| CT vs CC | 1.40(1.06-1.83) | **0.014** | 0.693 | **0.056** | **0.152** | 0.664 | 0.952 | 0.995 |
| CT+TT vs CC | 1.43(1.10-1.87) | **0.009** | 0.637 | **0.041** | **0.113** | 0.582 | 0.934 | 0.993 |
| Log-additive | 1.37(1.10-1.72) | **0.007** | 0.783 | **0.025** | **0.071** | 0.458 | 0.895 | 0.988 |
| **Age＞60** |  |  |  |  |  |  |  |  |
| CT vs CC | 1.49(1.02-2.18) | **0.040** | 0.514 | **0.189** | 0.412 | 0.885 | 0.987 | 0.999 |
| CT+TT vs CC | 1.58(1.09-2.29) | **0.016** | 0.392 | **0.107** | 0.265 | 0.799 | 0.976 | 0.998 |
| Log-additive | 1.54(1.12-2.12) | **0.008** | 0.436 | **0.053** | **0.143** | 0.648 | 0.949 | 0.995 |
| **male** |  |  |  |  |  |  |  |  |
| CT+TT vs CC | 1.44(1.04-2.00) | **0.030** | 0.596 | **0.130** | 0.309 | 0.831 | 0.980 | 0.998 |
| Log-additive | 1.43(1.08-1.90) | **0.014** | 0.629 | **0.061** | **0.163** | 0.682 | 0.956 | 0.995 |
| **Smoking-Yes** |  |  |  |  |  |  |  |  |
| CT+TT vs CC | 1.51(1.05-2.17) | **0.260** | 0.486 | **0.138** | 0.324 | 0.841 | 0.982 | 0.998 |
| Log-additive | 1.51(1.09-2.08) | **0.012** | 0.484 | **0.067** | **0.178** | 0.705 | 0.960 | 0.996 |
| **Drinking-Yes** |  |  |  |  |  |  |  |  |
| CT vs CC | 2.26(1.45-3.51) | **<0.001** | 0.034 | **0.024** | **0.070** | 0.452 | 0.893 | 0.988 |
| CT+TT vs CC | 2.29(1.48-3.52) | **<0.001** | 0.027 | **0.017** | **0.050** | 0.369 | 0.855 | 0.983 |
| Log-additive | 2.07(1.40-3.06) | **<0.001** | 0.053 | **0.015** | **0.043** | 0.330 | 0.832 | 0.980 |

FPRP: false positive report probability; SNP: single nucleotide polymorphism; OR: odds ratio; CI: confidence interval; CHD, coronary heart disease; HTN, hypertension; DM, diabetes mellitus.

The level of false-positive report probability threshold was set at 0.2 and noteworthy findings were presented.
